# Licorice extract and carbenoxolone protect PC12 cells against serum/glucose deprivation-induced apoptosis through modulation of caspase-3 and PARP activation

**DOI:** 10.22038/ajp.2024.25252

**Published:** 2025

**Authors:** Hossein Hosseinzadeh, Elham Ramazani, Soheyla Bakhshi, Zahra Tayarani-Najaran

**Affiliations:** 1 *Pharmaceutical Research Center, Pharmaceutical Technology Institute, Mashhad University of Medical Sciences, Mashhad, Iran*; 2 *Department of Pharmacodynamics and Toxicology, School of Pharmacy, Mashhad University of Medical Sciences, Mashhad, Iran*; 3 *Department of Biology, Yazd University, Yazd, Iran*; 4 *Medical Toxicology Research Center, Mashhad University of Medical Sciences, Mashhad, Iran.*; 5 *Targeted Drug Delivery Research Center, Pharmaceutical Technology Institute, Mashhad University of Medical Sciences, Mashhad, Iran*

**Keywords:** Licorice extract, Carbenoxolone, Apoptosis, Ischemia, PC12 cells

## Abstract

**Objective:**

Serum/glucose deprivation in cultured PC12 cells is considered an appropriate model for investigating detailed mechanisms of ischemia-induced brain injury. Here, we aimed to study the anti-apoptotic effects of licorice (*Glycyrrhiza glabra *L.) root extract and carbenoxolone on PC12 cells cultured in the serum/glucose deprivation (SGD) condition.

**Materials and Methods:**

Cells were incubated with the different concentrations of the *G. glabra *methanol extract (5-320 µg/ml) and carbenoxolone (0.5-32 µM) for 2 hr before being deprived of serum/glucose. Protection against cytotoxicity, increase in reactive oxygen species (ROS), and apoptosis was analyzed with resazurin, dichlorofluorescein diacetate (DCFH-DA), and western blot, respectively.

**Results:**

Serum/glucose deprivation induced cell death and apoptosis in PC12 cells. Pretreatment with the *G. glabra *methanol extract at 5-20 µg/ml and carbenoxolone at 0.5-2 µM for 2 hr significantly decreased the cytotoxicity (p<0.05), and pretreatment with the *G. glabra *methanol extract (5-160 µg/ml) and carbenoxolone (0.5 μM) significantly decreased the ROS content. Pretreatment with the *G. glabra *methanol extract and carbenoxolone at 5-20 µg/ml significantly prevented from the Poly (ADP-ribose) polymerase (PARP) and caspase-3 cleavage.

**Conclusion:**

Taken together, this study confirms the protective and free radical-scavenging potency of licorice extract and carbenoxolone in *in vitro* model of ischemia. Overall, it seems that pretreatment with the licorice extract and carbenoxolone may potentially slow the progression of brain ischemia.

## Introduction

One of the major causes of disability among elderlies worldwide is cerebrovascular diseases including focal cerebral ischemia (Parr, Ferdinand and Roffe 2017). Ischemia stroke has two types: thrombotic and embolic strokes that occur when a blockage by vascular thrombus formation or a rupture of a blood vessel cuts off the blood supply to the brain (Azami et al. 2021; Bradberry et al. 2004). Ischemia stroke reveals itself with various symptoms such as neurological deficits including hemiparesis, hemianaesthesia, aphasia, homonymous hemianopia and hemispatial inattention, motor impairments, seizures, and dementia (Azami et al. 2021; Campbell et al.). Also, ischemia stroke has been reported in young adults who are 55 years of age and younger because of the wider range of age-specific risk factors (Putaala 2020). Due to the high prevalence and incidence of ischemic stroke, several recent studies have offered natural antioxidant and anti-apoptotic products as potential remedies to combat oxidative stress and symptoms of ischemic stroke (Xie et al. 2021).

*Glycyrrhiza glabra *L. (Fabaceae) Licorice (U.S.)*/*liquorice (U.K.), is a Mediterranean region native plant with wide cultivation in Southeast Asia (India and Pakistan) and southern Europe (Akbar 2020; Asl and Hosseinzadeh 2008; Pastorino et al. 2018). As a flowering perennial plant, licorice has root and rhizome with prominent features, extensively used for culinary purposes, confectionery, and in folk and traditional medicine worldwide (Akbar 2020; Nassiri Asl and Hosseinzadeh 2007). Licorice is constituted of polysaccharides, flavonoids, isoflavones, triterpene, saponins, coumarins, stilbenoids, pectins, and miscellaneous compounds (Akbar 2020; Hosseinzadeh and Nassiri‐Asl 2015). All notable pharmacological effects of licorice on human health are attributed to the presence of multiple phytochemicals which act as antioxidant, antiulcer, anti-inflammatory, analgesic, antipyretic, antimicrobial, antiviral, antidepressant, antitumor, antidiabetic, antiallergic, and sedative agents (Akbar 2020; Ambavade, Kasture and Kasture 2001; Asl and Hosseinzadeh 2008; Cermelii et al. 1996; Ju et al. 1989; Lata et al. 1999; Pastorino et al. 2018; Yokota et al. 1998). Licorice has been potentially used to treat neurodegenerative disorders, gastrointestinal conditions, cardiovascular disorders, hepatotoxicity and liver damage, renal disorders, respiratory disorders (as antitussive), endocrine and skin diseases, atherosclerosis, immunodeficiency, hormone deficiency, and cancer (Asl and Hosseinzadeh 2008; Pastorino et al. 2018; Petramfar et al. 2020).

The major active triterpenoid saponin constituent of licorice root is glycyrrhizin (glycyrrhizic acid or glycyrrhizinic acid) which is 50× sweeter than sugar (Akbar 2020; Sarker and Nahar 2020).

 Carbenoxolone is a synthetic counterpart of glycyrrhizic acid, which has beneficial effects on oral ulcers, inflammation, and gastric ulcers and can increase insulin sensitivity (Asl and Hosseinzadeh 2008). Licorice and its derivatives are generally recognized as safe (GRAS) as a food supplement (Diomede et al. 2021). 

The molecular mechanisms underlying neural injury have recently been studied intensively. Apoptosis and overproduction of reactive oxygen species (ROS) have been proposed as important underlying mechanisms (Ekshyyan and Aw 2004; Lipton 1999; Won, Kim and Gwag 2002). Changes in the redox status of biomolecules present in neural cell membranes and the generation of ROS in hypoxic conditions alter the function of mitochondria and cause the activation of pro-apoptotic proteins. The PC12 cells are introduced as a model to study cerebral ischemia/reperfusion and neuronal protection *in vitro* (Hillion et al. 2005; Raya et al. 1993). Deprivation of cells from glucose, oxygen, and serum mimics the process called energy loss and neural injury resulted from cerebral ischemia/reperfusion. 

Although there are numerous reports on the pharmacologic properties of licorice root extract and carbenoxolone, the protective effects of the extract and its major component on neuron injury caused by ischemia have not yet been studied (Behravan, Razavi and Hosseinzadeh 2014). Taken together, the present study was designed to compare the neuroprotective effect of licorice root extract and carbenoxolones in an *in vitro* model of brain ischemia/neural injury on cultured PC12 cells starving from serum and glucose. Meanwhile, the role of the plant in protection against cleavage of caspase-3 and PARP was determined.

## Materials and Methods

### Plant material

The licorice (*G. glabra *L.) root was provided from an herbal store in Mashhad (Khorasan Razavi province, Northeastern of Iran). The plant was identified by Mrs. M. Souzani in the herbarium of the School of Pharmacy, Mashhad, Iran, and kept under standard situation.

### Preparation of the extracts

One hundred grams of the licorice root were soaked in 500 ml methanol for 24 hr mixture on a shaker and then filtered. Extraction and filtration were repeated three times. All filtrates were condensed with a rotary evaporator and dried at 40°C. The dried extract (8.9 g) was dissolved in dimethyl sulfoxide (DMSO). Phytochemical analysis of the plant extract was performed according to the De et al. study (De, Datta and Mukherjee 2012; Esmaeili et al. 2019; Varsha, Agrawal and Sonam 2013). 

### Cell culture and treatment

PC12 cells obtained from the National Cell Bank of Iran (NCBI) and cultured in standard conditions contained high glucose Dulbecco’s Modified Eagle’s Medium (DMEM) (4.5 g/L) plus fetal bovine serum (FBS-10% v/v), 100 U/ml penicillin, and 100 µg/ml streptomycin at 37°C with 90% humid and 5% CO_2_. A control sample was an untreated cell with an equal volume of solvent in the culture medium (Emami et al. 2019; Naserian et al. 2018).

To evaluate the effects of the licorice extract and carbenoxolone against serum/glucose deprivation in PC12 cells, 2 hr pretreatment of cells with different concentrations of *G. glabra *methanol extract (5-320 µg/ml) and carbenoxolone (0.5-32 μM) was selected. After 6 hr serum/glucose-free DMEM, the viability and ROS content were determined. Western blot analysis was done on cells pretreated with 5-20 µg/ml of *G. glabra *methanol extract and 0.5-2 μM of carbenoxolone for 18 hr before exposure to serum/glucose deprivation (SGD) stress (Hadipour, Tayarani-Najaran and Fereidoni 2020).

### Cell viability

Resazurin (AlamarBlue^®^) is reduced to resorufin when exposed to live cells (O'brien et al. 2000). PC12 cells (4×10^3^ cells per well) in 96-well plates were treated as described previously. After 6 hr incubation, the viability was quantified with resazurin reagent (20 μl; 10 mg/ml) comparing the absorbance of 600 nm read by ELISA microplate reader (Awareness, Palm City, FL, USA) (Tayarani-Najaran et al. 2021).

### Intracellular ROS analysis

DCFH-DA (2′,7′-dichlorofuorescin diacetate) reagent is a cell-permeable non-fluorescent probe which is used to detect oxidative products in various cells (Chen et al. 2010). PC12 cells (4×10^3^ cells per well) were cultured in 96-well plates and treated as described previously. After an additional 2 hr, cells were treated with 5 µM DCFH-DA at 37°C in the dark for 30 min. Then, ROS generation was measured and compared to the related control after assessment with the micro plate fluorimeter (excitation wave length, 485 nm, and emission wave length, 530 nm) (Paradigm multi- mode plate reader; Becton, Dickinson and Company, Franklin Lakes, NJ, USA) (Taheri, Hadipour and Tayarani-Najaran 2021).

### Western blot analysis

Regarding the protocol of our previous study (Rezazadeh‐Shojaee et al. 2022), western blot analysis was used to detect the expression of caspase-3 and poly ADP ribose polymerase (PARP) protein levels. After 18 hr incubation, treated PC12 cells (10^6^ cells) were washed with cold PBS (4°C) followed by protein extraction. Ultimately, the levels of caspase-3 and PARP proteins were normalized according to their corresponding β-actin band (Gel Doc UV Alliance, Alliance 4.7, UK).

### Statistical analysis

The one‐way analysis of variance (ANOVA) and the Tukey–Kramer post hoc test were used to evaluate the differences between the groups. All the results are expressed as mean±SEM and *p* values below 0.05 were considered statistically significant. Each experiment was repeated at least three times.

## Results

### Effects of the G. glabra methanol extract and carbenoxolone on cytotoxicity induced by SGD stress in PC12 cells

**Figure 1:**
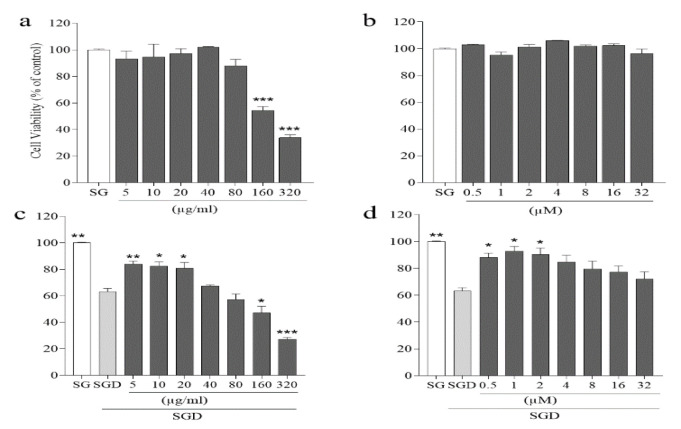
*Cell* *viability: (a) Effects of various concentration (5-320 µg/ml) of the *G. glabra* methanol extract on the viability of PC12 cells, (b) Effects of various concentrations (0.5-32 μM) of carbenoxolone on the viability of PC12 cells, (c) Effect of various concentrations of the* G. glabra* methanol extract (5-320 µg/ml) for 2 hr on PC12 cells before exposure* *to SGD (serum/glucose deprivation) stress for 6 hr, and (d) Effect of various concentrations of carbenoxolone (0.5-32 μM) for 2 hr on PC12 cells before exposure* *to SGD stress for 6 hr. Pretreating cells with the *G. glabra methanol extract (5-20 µg/ml) and carbenoxolone (0.5-2 μM) significantly could prevent SGD-induced cytotoxicity. SG: Control cells received high glucose (4.5 g/ml) DMEM supplemented with FBS. Values are the mean±SEM of three independent experiments in triplicate. *p<0.05, **p<0.01, and ***p<0.001 compared to SGD-stress group.

First, the optimum cytotoxic concentration (CC 50) was determined to evaluate the cytotoxicity and assessment of the protective effects of *G. glabra *methanol extract and carbenoxolone. Based on the results, when PC12 cells were exposed to SGD stress for 6 hr, cell viability significantly decreased by 63.2±4.0% (p<0.001) compared to untreated cells (Fig. 1a). After that, the cytotoxicity of *G. glabra *methanol extract (5-320 µg/ml) and carbenoxolone (0.5-32 μM) was measured. Cell viability of PC12 cells has shown no significant changes compared to untreated cells when cells were incubated with the *G. glabra *methanol extract (5-80 µg/ml) and carbenoxolone (0.5-32 μM) (Figure 1a and b). When PC12 cells were treated with *G. glabra *methanol extract (5-80 µg/ml) and carbenoxolone (0.5-32 μM) 2 hr before SGD stress exposure, *G. glabra *methanol extract (5-20 µg/ml) and carbenoxolone (0.5-2 μM) significantly attenuated SGF-induced cytotoxicity in PC12 cells (p<0.05) (Figure 1c and d). 

### Effects of the G. glabra methanol extract and carbenoxolone on SGD-induced ROS generation in PC12 cells

**Figure 2:**
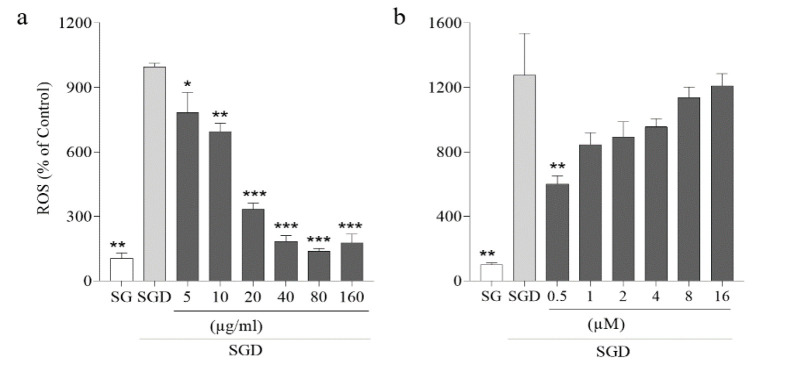
*Fluorimetry with DCFH-DA staining for measuring ROS production: (a) Effects of various concentrations of the* G. glabra* methanol extract (5-160 µg/ml) for 2 hr on intracellular ROS in PC12 cells induced by SGD (serum/glucose deprivation) stress for 6 hr, and (b) Effects of various concentrations of carbenoxolone (0.5-16 μM) for 2 hr on intracellular ROS in PC12 cells induced by SGD stress for 6 hr. Pretreating cells with the *G. glabra methanol extract (5-160 µg/ml) and carbenoxolone (0.5 μM) significantly could prevent the ROS increment induced by SGD-stress. SG: Control cells received high glucose (4.5 g/ml) DMEM supplemented with FBS. Values are the mean±SEM of three independent experiments in triplicate. *p<0.05, **p<0.01 and ***p<0.001 compared to SGD-stress group.

Alterations in intracellular ROS levels were detected with fluorimetry by molecular probe DCFH-DA (Chen et al. 2010). Based on the results, incubation of PC12 cells with SGD stress for 2 hr showed a significant increase in intracellular ROS levels. However, cell pretreatment with the *G. glabra *methanol extract (5-160 µg/ml) and carbenoxolone (0.5 μM) (p<0.01) significantly attenuated the ROS production following SGD stress and exhibited a protective effect against SGD-induced cytotoxicity (Figure 2). 

### Effects of the G. glabra methanol extract and carbenoxolone on SGD-induced cell death based on Western blot analysis

**Figure 3:**
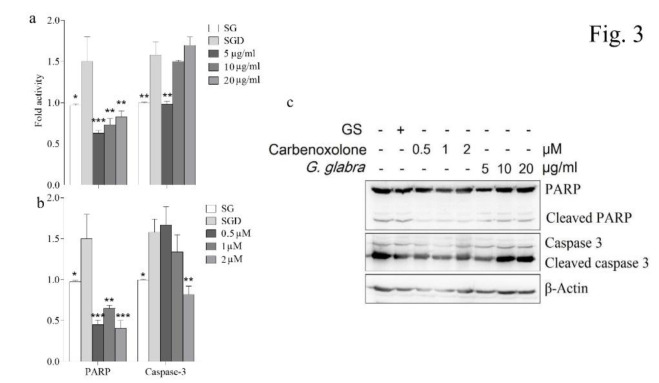
Effect of the *G. glabra *methanol extract (5-20 µg/ml) and carbenoxolone (0.5-2 μM) pretreatment on protein levels of PARP and caspase-3 as assessed by western blot analysis in PC12 cells induced by SGD (serum/glucose deprivation) stress. (a) Pretreatment with the *G. glabra *methanol extract at 5-20 µg/ml significantly decreased cleaved PARP (p<0.05), and at 5 µg/ml significantly decreased cleaved caspase-3 (*p*<0.01) levels to a level near that of control, and (b) Pretreatment with carbenoxolone (0.5-2 μM) significantly decreased cleaved PARP, and at 2 μM significantly decreased cleaved caspase-3 (*p*<0.01) levels to a level near that of control. (c) The western blotting image, (SG: control cells received high glucose (4.5 g/ml) DMEM supplemented with FBS). Values are the mean±SEM of three independent experiments in triplicate. *p<0.05, ** p<0.01, and ***p<0.001 compared to SGD-stress group.

Recent studies recommend that SGD stress could activate some main modulators of apoptosis to initiate the cellular apoptosis pathways (Abdolmaleki, Ghayour and Behnam-Rassouli 2020; Tang et al. 2015). In the present study, pretreatment with the *G. glabra *methanol extract at 5-20 µg/ml significantly decreased cleaved PARP (p<0.05), and at 5 µg/ml significantly decreased cleaved caspase-3 (*p*<0.01) levels to a level near that of control. Also, pretreatment with carbenoxolone (0.5-2 μM) significantly decreased cleaved PARP, and at 2 μM significantly decreased cleaved caspase-3 (p<0.01) levels to a level near that of control (Figure 3).

## Discussion

Oxidative stress is known as a key factor for the pathogenetic process and mechanism of ischemic brain damage. Generating oxidative stress results in the brain tissue damage and subsequent cellular apoptosis (Orellana-Urzúa et al. 2020). Also, excessive ROS production oxidizes and modifies many biological macromolecules such as proteins, lipids, and DNA, which leads to apoptosis in neurodegenerative diseases (Luceri et al. 2018). Therefore, recent studies have turned more toward using plant-derived products with neuroprotective functions to prevent or diminish adverse pathological effects of neurodegenerative diseases such as brain ischemia. Licorice is used throughout the world as a traditional medicine and food industry ingredient (Ekshyyan and Aw 2004; Won, Kim and Gwag 2002). This plant has revealed numerous therapeutic properties, including antioxidant, anti‐inflammatory, antiviral, antimicrobial, anticancer, and hepatoprotection effects (Moustafa, El-Azab and Fouda 2013; Won, Kim and Gwag 2002). In addition, licorice root is suggested for microbial/viral infection, cancer, and skin diseases (Moustafa, El-Azab and Fouda 2013). Based on the results of phytochemical analysis, the hydro-methanolic extracts of *G. glabra* consist of saponin, flavonoids, alkaloids, steroids, terpenoids, tannins and glycosides (Varsha, Agrawal and Sonam 2013). Glycyrrhizic acid (GA), glabridin (GB), liquiritin (LQ) and liquiritigenin (LG) are flavonoids in licorice root based on the HPLC chromatogram (Esmaeili et al. 2019). Numerous studies reported various pharmacological effects of glycyrrhizic acid such as anti-inflammatory, anti-hepatotoxic, stimulant, depletive, anti-gastric ulcer, and antivirus effects (Esmaeili et al. 2019). 

Carbenoxolone is a widely-investigated synthetic derivative of licorice root that possesses neuroprotective properties by affecting the apoptosis signaling pathways (Wang et al. 2015; Zhang et al. 2013). The SGD‐induced apoptosis in PC12 cells was manipulated to evaluate and compare the protective effects of the *G. glabra *methanol extract and carbenoxolone in an *in vitro *model of brain ischemia. 

The present study showed that pretreatment with the *G. glabra *methanol extract (5-20 µg/ml) and carbenoxolone (0.5-2 μM) significantly increased PC12 cell viability. Additionally, pretreatment with *G. glabra *methanol extract (5-160 µg/ml) and carbenoxolone (0.5 μM) significantly decreased the ROS amount. Also, decreases in the level of cleaved PARP and cleaved caspase-3 protein were all detected with *G. glabra *methanol extract and carbenoxolone in SGD-induced PC12 cells. The results showed that the *G. glabra *methanol extract and carbenoxolone significantly inhibited the activation of the PARP and caspase-3 in PC12 cells exposed to SGD stress. Although the anti-apoptotic activities of the *G. glabra *methanol extract and carbenoxolone were modest, it seems when *G. glabra *methanol extract and carbenoxolone are used *in vivo* it may attenuate the activity of apoptosis signaling pathways in the body.

Few researches reported the antioxidant and anti-apoptosis features of *G. glabra *and carbenoxolone. In a study implemented in 2015, carbenoxolone showed its anti-apoptotic activities via PI3K/Akt pathways and suppressing the caspase-3 apoptosis pathway in rats exposed to transient focal ischemia and reperfusion (I/R) (Zhang et al. 2013). In another study on the effect of methanol extract of licorice on the brains of middle cerebral artery occlusion (MCAO)-induced mice (as a model of thrombotic stroke), it was shown that pretreatment with methanol extract of licorice exhibited anti-apoptotic properties. The anti-apoptotic mechanisms of licorice are mainly associated with the overexpression of anti-apoptotic bcl-2 and bcl-xL, and the reduction of the changes affected the expression of caspase-9 proteins (Lim et al. 2018). Similarly, Lee et al. have found that methanol extract of *Glycyrrhizae Radix* has a significant effect on the volume of the infarcted area and cell survival in the MCAO mouse model (Lee et al. 2018). Also, 5 days of treatment with 20 mg/kg glycyrrhizic acid noticeably reduced the level of lipid peroxidation and increased the activity of superoxide dismutase, and showed antioxidant properties in a vascular dementia (VD) rat model (Guo et al. 2016). The neuroprotective effects of carbenoxolone in the MCAO rat as an *in vivo* model of cerebral ischemia and in H_2_O_2_- treated PC12 cells an *in vitro* model of hypoxic-ischemic brain were examined in 2013. Results of the *in vivo* study indicated that intracerebroventricular of injection 25 mg/kg carbenoxolone 30 min before cerebral ischemic surgery decreased the expression of connexin 43 (Cx43) in the ipsilateral striatum, ROS generation, and activation of microglia and astrocytes in MCAO rat. Based on the *in vitro* results, carbenoxolone (100 and 200 μM) inhibited the opening of gap junctions, Cx43 expression, and apoptosis ratio and enhanced the cell viability in H_2_O_2_- treated PC12 cells (Zhang et al. 2013). Gap junctions remain open after cerebral ischemia, and it has been reported that the phosphorylation of Cx43 in ischemic stroke might induce the degradation of gap junctions and the opening of hemichannels (Cotrina et al. 1998; Liang et al. 2020; Rami, Volkmann and Winckler 2001; Rawanduzy et al. 1997; Thompson, Zhou and MacVicar 2006). In a rat model of ischemia/reperfusion (I/R) in the hind limb and hippocampus, administration of 100-200 mg/kg of carbenoxolone after reperfusion significantly diminished the changes observed in malondialdehyde (MDA) levels and showed anti-oxidant effects (Hosseinzadeh, Asl and Parvardeh 2005). According to Vakili et al. (2009) peripheral administration (100, 200, or 400 mg/kg, intraperitoneally) of carbenoxolone at the beginning of ischemia significantly restrained cortical infarct volumes, and striatal infarct volumes in a rat model of transient cerebral ischemia (Vakili, Hosseinzadeh and Khorasani 2009). Interestingly, administration of carbenoxolone (1, 12, 25, and/or 50 μg/kg) into the right ventricle at the beginning of the MCAO rat model remarkably reduced cortical infarct volumes, also at 25 μg/kg significantly decreased the infected volume of striatal and combat against neurological dysfunctions (Khorasani, Hosseinzadeh and Vakili 2009).

**Figure 4:**
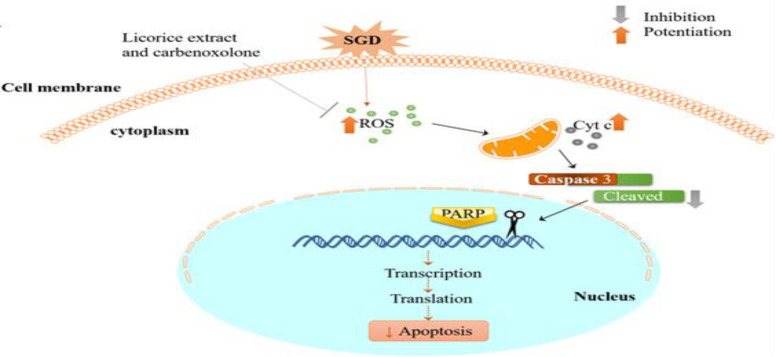
*The protective mechanisms of the *G. glabra* methanol extract and carbenoxolone in reducing SGD-induced cytotoxicity to PC12 cells.*

In the case of the role of apoptosis in neurodegeneration, it has been proposed that alteration in the amount or change in the activity of apoptotic protein may also be related to the antioxidant capacity of the compound (Mousavi, Tayarani and Parsaee 2010; Uttara et al. 2009). The apoptosis cascade may be blocked either through the blockage of the intrinsic or extrinsic mediators involved in the process. Both pathways merge in caspase-9 activation, which then cleaves the DNA repairing enzymes like PARP. While caspase-8 is the mediator of the external pathway, caspase-9, cyt-c and bcl-2 family are internal pathway mediators (Kreuzaler and Watson 2012). Since mitochondrion is a source of ATP for the cell it has a pivotal role in maintaining the cell vitality. Limitation in glucose and oxygen impairs oxidative metabolism affects the cell and causes ischemic degeneration. In ischemic neurons, mitochondria release apoptotic mediators, which contribute to cell damage (Sims and Muyderman 2010). According to our results, *G. glabra* and carbenoxolone both showed anti-oxidant activity via diminishing ROS amount and protected against SGD-induced cytotoxicity. We demonstrated that *G. glabra* and carbenoxolone both are capable of suppressing the PARP and caspase-3 activities in PC12 cells exposed to SGD stress. Overall, the synergistic effects of the phytochemicals in *G. glabra *methanol extract with carbenoxolone may potentiate the neuroprotective effect of licorice against brain ischemia (Figure 4)

To sum up, we concluded that *G. glabra *methanol extract and carbenoxolone could protect PC12 cells against SGD‐induced toxicity inhibition of apoptosis and the cleavage of PAPR and caspase-3. To conclude, it seems *G. glabra* and carbenoxolone both can protect neural cells with antioxidant and anti-apoptotic effects and may potentially reduce the progression of brain ischemia.

## Abbreviations

AO/EB: Acridine orange/ethidium bromide; ANOVA: Analysis of variance; Bcl-2: b-cell lymphoma 2; Bcl-xL: bcl extra-large; CNS: Central nervous system; Cyt c: Cytochrome c; Cx43: Connexin 43; DMEM: Dulbecco’s Modified Eagle’s Medium; ER: Endoplasmic reticulum; FBS: Fetal bovine serum; FDA: Food and drug administration; GRAS: Generally recognized as safe; GRex: Glycyrrhizae Radix et Rhizome; GSD: Serum/glucose deprivation; JECFA: Joint FAO/WHO Expert Committee on Food Additives; MCAO: Middle cerebral artery occlusion; MDA: Malondialdehyde; NO: Nitric oxide; ROS: Reactive oxygen species; PARP: poly ADP ribose polymerase; PBS: phosphate-buffer saline; PGs: Prostaglandins; VD: vascular dementia.
